# Design and Synthesis of an Azo Reductase Responsive Flavonol–Indomethacin Hybrid Used for the Diagnosis and Treatment of Colitis

**DOI:** 10.3390/molecules29174244

**Published:** 2024-09-06

**Authors:** Yaqin Gu, Rui Yang, Jine Chen, Yu Fan, Wenna Xie, Hongyan Wu, Jinfeng Ding

**Affiliations:** 1 School of Pharmacy, Jiangsu Vocational College of Medicine, Yancheng 224005, China; guyaqin@jsmc.edu.com (Y.G.); 12359@jsmc.edu.cn (R.Y.); eee214@sina.com (J.C.); xiewenna_ch@163.com (W.X.); wuhongyan@jsmc.edu.cn (H.W.); 2 School of Pharmacy, Nantong University, Nantong 226019, China; fanyu_1998@sina.com

**Keywords:** ulcerative colitis, azoreductase, flavonols, indomethacin, diagnosis, treatment

## Abstract

Human intestinal bacteria are the primary producers of azo reductase, and the content of azo reductase is closely associated with various intestinal diseases, including ulcerative colitis (UC). The rapid detection of changes in azo reductase levels is crucial for diagnosing and promptly intervening in UC. In this study, a therapeutic agent, **FAI**, specifically targeting UC, was designed and synthesized. This agent was developed by linking the anti-inflammatory drug indomethacin to flavonols with antioxidant activity via an azo bond (off–on). Breakage of the azo bond breaks results in the release of both fluorophores and drugs, achieving targeted tracing and integrated treatment effects. In vivo and in vitro fluorescence imaging experiments were used to demonstrate the potential of **FAI** in the diagnosis of UC, together with synergistic therapeutic effects through the release of both fluorophores and anti-inflammatory agents. Therefore, this diagnostic agent shows promise as a potential tool for diagnosing and treating UC.

## 1. Introduction

Ulcerative colitis (UC) is a chronic inflammatory bowel disease with an unknown pathogenesis, and its incidence is rising worldwide. UC has a prolonged course, is challenging to treat, and may progress to more severe conditions, such as colorectal cancer, posing a significant economic and medical burden [1,2,3]. Currently, endoscopic biopsy is the only definitive diagnostic method for UC [4]. Therefore, early detection and timely intervention are of great clinical importance.

Azo reductase is a flavin-dependent enzyme expressed in various prokaryotic and eukaryotic organisms, and it is commonly referred to as flavin mononucleotide reductase [5]. This enzyme efficiently reduces arylazo (N=N) compounds to aromatic amines. It is found primarily in tumors and the intestines, linking it closely to diseases, such as cancer and UC [6,7,8,9,10,11]. Azo reductase is widely utilized in disease diagnosis and drug delivery. Azo groups are frequently incorporated to create off–on probes that specifically recognize azo-reducing enzymes for diagnostic purposes. Furthermore, azo groups can function as targeting moieties for colitis, where drug molecules are released in situ upon reduction by azo-reducing enzymes at the colon site. This mechanism allows for the highly effective targeted and controlled release of drugs [12,13,14,15,16,17,18], such as sulfasalazine (SASP), olsalazine, and sulphasalazine, which contain azo-targeting groups in their molecular structures. Although some tumor diagnostic and therapeutic agents based on azo bond design have been reported [19,20,21], there have been few investigations into their applications in the diagnosis and treatment of UC.

Flavonols are important natural compounds characterized by the chemical structure 3-hydroxy-2-phenyl-1-benzopyran-4-one. They are commonly found in flowers, fruit, vegetables, and traditional Chinese medicine and exhibit various pharmacological activities, including antioxidant [4], anti-inflammatory [22,23], antibacterial [24,25], antiallergic [26], anticancer [27,28], and antiviral [29] activities. Moreover, flavonols are also used as natural plant dyes due to their intrinsic excited-state intramolecular proton transfer (ESIPT) properties, associated with excellent photostability and high fluorescence quantum yields. As a result, flavonols are regarded as promising fluorescent agents with potential applications in biological fields.

**Scheme 1:**
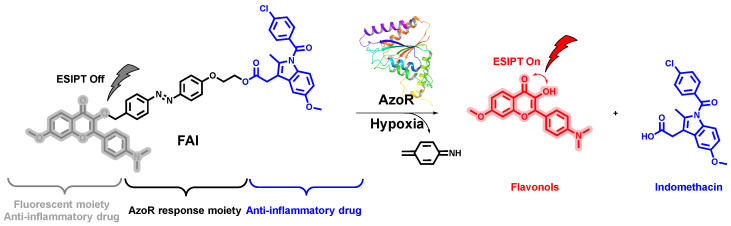
Schematic diagram of releasing indomethacin and azo bond cleavage from diagnosis and treatment agents (**FAI**) for fluorescence emission “turn on”.

To facilitate the early diagnosis and prompt treatment of UC, the present study focused on the synthesis and biological assessment of a novel prodrug termed **FAI**, which is responsive to azo reductase. This therapeutic compound incorporates a flavanol component and indomethacin (INDO), functioning synergistically, to enhance the diagnosis and treatment of UC (Scheme 1).

## 2. Results and Discussion

### 2.1. Chemistry

**Scheme 2:**
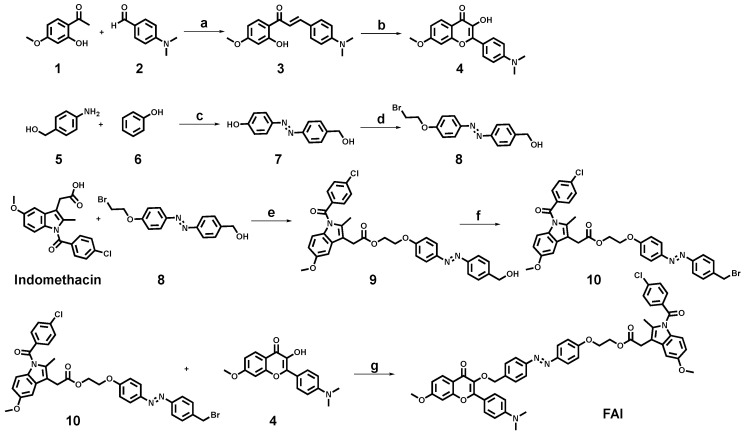
Synthesis of the target compound **FAI**. Reagents and conditions: (**a**) 20% KOH, EtOH, r.t., overnight; (**b**) 5% NaOH, H_2_O_2_, EtOH, r.t., 4 h; (**c**) NaNO_2_, conc. HCl, H_2_O, K_2_CO_3_, 0 °C, 2 h; (**d**) 1,2-dibromoethane, K_2_CO_3_, acetone, 65 °C, 8 h; (**e**) K_2_CO_3_, acetone, 65 °C, 8 h; (**f**) CBr_4_, PPh_3_, THF, r.t., 3 h; (**g**) K_2_CO_3_, DMF, 65 °C, 8 h.

The synthesis of the compound **FAI** is outlined in Scheme 2. Chalcone (**3**) was synthesized using the Claisen–Schmidt condensation method. Briefly, acetophenone (**1**) and an aromatic aldehyde (2) were condensed and dehydrated in the presence of a dilute solution of aqueous sodium hydroxide, yielding chalcone **3** with a yield of 68%. Chalcone 3 then underwent cyclization through the Algar–Flynn–Yamada reaction under alkaline conditions in the presence of H_2_O_2_, resulting in Compound **4** with a moderate yield of 52% due to concurrent side reactions. Compound **7** was synthesized in a similar manner, resulting in a yield of 92%, through the azo coupling reaction between 4-aminobenzyl alcohol and phenol using NaNO_2_ under mildly acidic conditions. The phenolic hydroxyl group of azo Compound **7** then reacted with dibromoethane in the presence of K_2_CO_3_, resulting in the formation of Compound **8** (82% yield). The carboxylic group of indomethacin was esterified with Compound **8** in the presence of K_2_CO_3_, resulting in the formation of Compound **9** (85% yield). The alcohol hydroxy group of Compound **9** was then transformed into a bromo-hydrocarbon through the Appel reaction, catalyzed by CBr_4_ and PPh_3_, yielding Compound **10** with a 73% yield. Subsequently, flavonoidol (**4**) reacted with the indomethacin-azo complex (**10**) in the presence of K_2_CO_3_, leading to the synthesis of the target compound **FAI** with a yield of 79%.

### 2.2. Photophysical Properties of Compound ***4*** 

**Figure 1:**
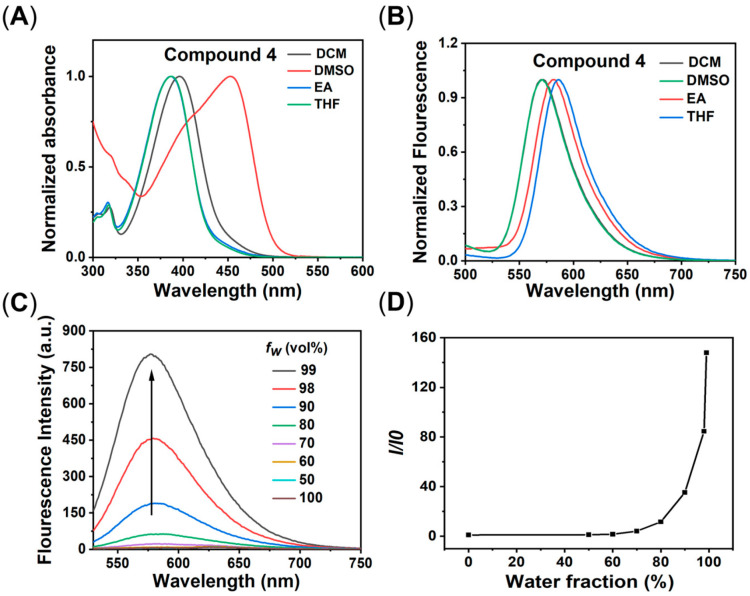
(**A**) UV absorption spectra of Compound **4** (20 μM) in different solvents (DCM, DMSO, EA, and THF); (**B**) fluorescence spectra of Compound **4** (20 μM) in different solvents (DCM: λ_ex_ = 400 nm, DMSO: λ_ex_ = 450 nm, EA: λ_ex_ = 390 nm and THF: λ_ex_ = 390 nm, slit: 5 nm/5 nm); (**C**) changes in the fluorescence spectra of Compound **4** (20 μM) in DMSO/H_2_O (*fw*) solutions with different water contents; (**D**) plots of I/I_0_ values for Compound **4** in DMSO/H_2_O mixtures with different *fw* values.

To explore the photophysical characteristics of Compound **4**, UV-VIS absorption and fluorescence spectra were first acquired and analyzed using various solvents. The concentration of probe **4** was maintained at 20 μmo/L using solvents, including dichloromethane (DCM), dimethyl sulfoxide (DMSO), ethyl acetate (EA), and tetrahydrofuran (THF). As depicted in Figure 1A, probe **4** exhibited an absorption peak around 400 nm (approximately 450 nm in DMSO), attributed to an intramolecular charge transfer transition. In the fluorescence emission spectra (Figure 1B), the excitation wavelength corresponded to the maximum absorption wavelength of each solvent, with probe **4** displaying a consistent maximum emission wavelength of approximately 570 nm across all four solvents, indicating a Stokes shift of 170 nm. The molar absorption coefficient of Compound **4** at 450 nm in the DMSO solution was 27,400 M^−1^ cm^−1^, and the absolute fluorescence quantum yield was 13.6% (Figure S11 and Table S1). The emission spectra of the probes were further investigated in various volume fractions (*fw*) of DMSO/H_2_O mixtures (Figure 1C). Initially, as the moisture content increased, the emission intensity remained relatively stable. However, when the concentration was raised to above 80%, the fluorescence intensity increased gradually (Figure 1D), confirming the presence of the aggregation-induced emission (AIE) characteristics of this probe.

### 2.3. Release Properties of Compound ***FAI*** 

Given the exceptional optical properties of **FAI**, an additional investigation was carried out to explore the effects of its fluorescence “off–on” behavior and drug-release characteristics on catalysis by azo reductase.

**Figure 2:**
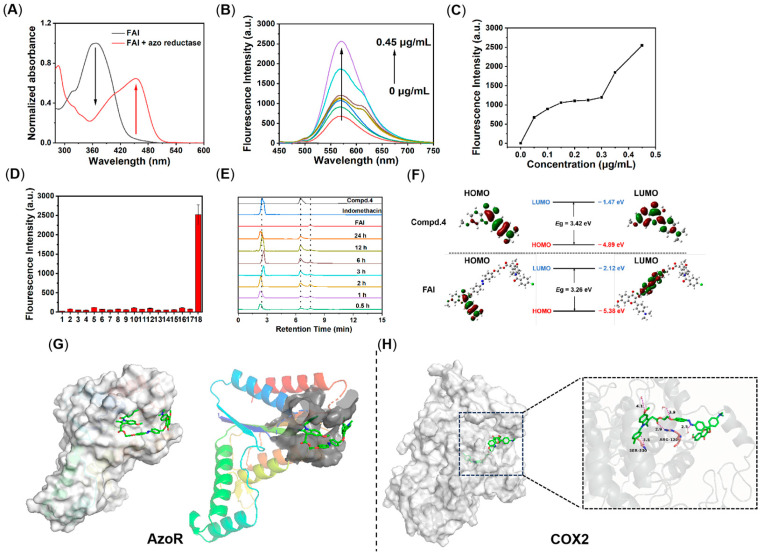
Release of compounds from **FAI** by azo reductase. (**A**) UV absorption spectra of **FAI** (20 μM) in the presence and absence of azo reductase (0.4 μg/mL). (**B**) Fluorescence emission spectra of **FAI** (20 μM) at different concentrations of azo reductase (0–0.45 μg/mL). (**C**) The fluorescence intensity of **FAI** at 578 nm changed in response to the enzyme concentration. (**D**) Intensities of the fluorescence response in the presence of different analytes (1, blank; 2, K^+^; 3, Na^+^; 4, Mg^2+^; 5, ClO^−^; 6, Ca^2+^; 7, GSH; 8, Zn^2+^; 9, Fe^2+^; 10, alkaline phosphatase (ALP); 11, Cys; 12, OH^−^; 13, VcNa; 14, Cu^2+^; 15, NADPH; 16, glucose; 17, H_2_O_2_; 18, azo reductase) of **FAI** (20 μM). (**E**) HPLC analysis of **FAI** (50 μM) incubated with azo reductase (0.4 μg/mL) at different time points (0.5, 1, 2, 3, 6, 12, and 24 h). (**F**) The frontier molecular orbitals of Compound **4** and **FAI**. (**G**) Docking simulation showing **FAI** within the active site of azo reductase (PDB code: 2Z9C). (**H**) Docking simulation showing **FAI** within the binding site of COX2 (PDB code: 4OTJ).

Initially, the absorption spectra of **FAI** were investigated in a buffer solution (pH 7.4, 1% DMSO) in the presence and absence of azo reductase and NADH. As depicted in Figure 2A, **FAI** exhibited a maximum absorption peak centered around 360 nm. This peak decreased with the addition of azo reductase, and a new peak emerged at 450 nm, indicating changes in the molecular structure. The fluorescence spectrum, illustrated in Figure 2B, demonstrated that the ESPIT effect of flavonol was disrupted by the azobenzene group, significantly reducing the fluorescence emission from the probe. In the presence of azo reductase, the fluorescence intensity at 570 nm increased in a concentration-dependent manner (Figure 2C), reaching a maximum at 0.45 μg/mL. A comparison with the fluorescence spectrum of Compound **4** showed that the fluorescence recovered to the original 90.6% after cleavage. Next, the incubation of **FAI** with different physiologically relevant substances, including K^+^, Na^+^, Mg^2+^, ClO^−^, Ca^2+^, GSH, Zn^2+^, Fe^2+^, alkaline phosphatase (ALP), Cys, OH^−^, VcNa, Cu^2+^, NADPH, glucose, H_2_O_2_, and azo reductase, was used to evaluate its selectivity for azo reductase. As shown in Figure 2D, the fluorescence intensity of **FAI** changed following the treatment with azo reductase. In contrast, the incubation of **FAI** with other biologically relevant species resulted in only a minimal increase in fluorescence. This finding suggests that **FAI** responds specifically to azo reductase, leading to drug release.

Moreover, the kinetics of Compound **4** and indomethacin release from **FAI** when exposed to azo reductase were investigated over 24 h. The HPLC chromatogram (Figure 2E) shows the initial appearance of a retention peak for **FAI** at 7.5 min. Two additional retention peaks subsequently emerged at 2.5 min (indomethacin) and 6.4 min (Compound **4**), indicating the enzyme-mediated release of both compounds from **FAI**. After 24 h of incubation, the complete release of both Compound **4** and indomethacin was observed. These findings suggest that **FAI** releases both Compound **4** and indomethacin upon exposure to azo reductase, thereby facilitating synergistic therapeutic effects.

Scheme 2 proposes a possible response mechanism. The non-fluorescent nature of the probe itself is attributed to the disruption of the ESPIT effect between the 3-hydroxy and 4-carbonyl groups of Compound **4** by the azobenzene group. When exposed to azo reductase, the reduction of the azobenzene group leads to the cleavage of Compound **4** and indomethacin, facilitating the ESPIT process and resulting in the generation of a strong fluorescent signal. To gain deeper insight into this mechanism, density functional theory (DFT) calculations were performed using Gaussian 09 software. The highest occupied molecular orbital (HOMO) and lowest unoccupied molecular orbital (LUMO) of Compound **4** and **FAI** are shown in Figure 2F. The LUMO energy level of **FAI** indicates that the charge distribution primarily resides within the azobenzene group, confirming its role in disrupting the ESPIT process.

To investigate the binding interactions between **FAI** and azo reductase and COX-2, molecular docking was performed using Schrödinger–Maestro. The results show that the azo group of **FAI** fit snugly within the active pocket of azo reductase. Meanwhile, indomethacin was shown to enter the active pocket of COX-2 and form hydrogen bonds with amino acid residues Ser-530 and Arg-120. These experimental findings support the proposed response mechanism.

### 2.4. In Vitro Anti-Inflammatory Activity

Treatment with LPS activates macrophages, resulting in the generation of substantial amounts of nitric oxide (NO), which can exacerbate the progression of inflammatory diseases. Hence, NO production in LPS-treated RAW 264.7 mouse macrophages was evaluated to assess the inhibitory effects of Compound **4**, indomethacin, Compound **4** + indomethacin, and **FAI**. The results are shown in Table 1, indicating that **FAI** demonstrated significant anti-inflammatory activity, with an IC_50_ value of 5.1 ± 0.48 μM. This efficacy surpassed that of Compound **4** and indomethacin when used individually and was comparable to their combined activity. Notably, analysis of the cytotoxicity revealed that **FAI** significantly mitigated the toxicity associated with indomethacin when used alone.

**Figure 3:**
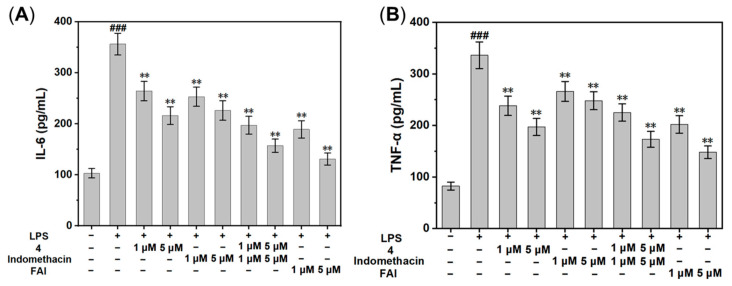
Effects of Compound **4**, indomethacin, **4** + indomethacin, **FAI** (1 or 5 μM) on IL-6 (**A**) and TNF-α (**B**) expression in LPS-induced RAW 264.7 cell by ELISA. Data are expressed as the mean ± SD (*n* = 3). ### *p* < 0.01 compared with the control group; ** *p* < 0.01 compared with the LPS group.

The production of the inflammatory cytokines TNF-α and IL-6 by macrophages after **FAI** treatment was measured using ELISA. As seen in Figure 3A,B, treatment with all compounds at 1 µM resulted in significantly reduced inflammatory cytokine secretion. This inhibition of inflammatory cytokine secretion was most marked at an **FAI** concentration of 5 µM, underscoring its pronounced anti-inflammatory efficacy.

### 2.5. Antioxidant and Anti-Mitochondrial Damage

**Figure 4:**
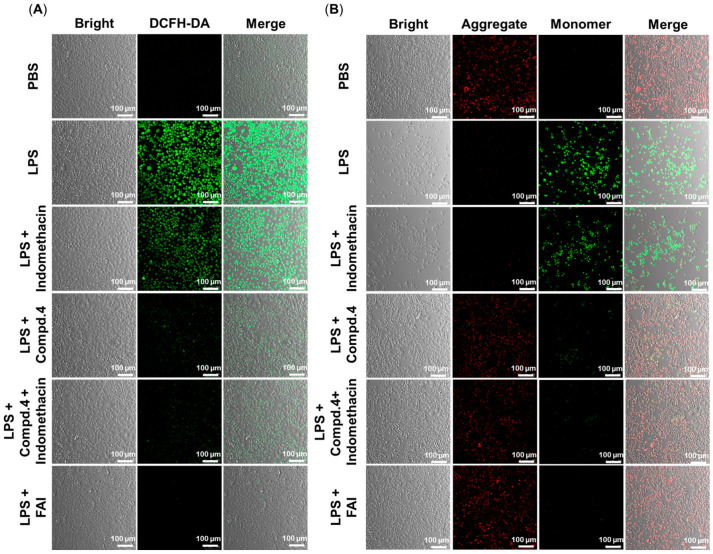
(**A**) The influence of **FAI** on ROS levels resulting from the LPS treatment of RAW264.7 cells. The ROS levels were determined using DCFH-DA in cells treated with indomethacin, Compound **4**, a combination of indomethacin and Compound **4**, and **FAI**, as shown by laser confocal microscopy. λex = 488 nm; λem = 500–550 nm; scale bar = 100 μm. (**B**) The influence of **FAI** on the mitochondrial membrane potential in LPS-treated RAW264.7 cells. Changes in the mitochondrial membrane potential were determined by JC-1 accumulation in cells treated with indomethacin, Compound **4**, a combination of indomethacin and Compound **4**, and **FAI**, as shown by laser confocal microscopy. Green channel: λex = 488 nm, λem = 510–550 nm; red channel: λex = 535 nm, λem = 590–610 nm; scale bar = 100 μm.

Recent research shows a strong association between mitochondrial oxidative stress and UC [30,31]. Figure 4A illustrates the impact of **FAI** on the levels of reactive oxygen species (ROS) in LPS-induced RAW264.7 cells. Cells in the model group showed markedly increased intracellular ROS levels in comparison to the blank control group. Compound **4** significantly reduced the intracellular ROS levels, in contrast to indomethacin, which showed limited efficacy. Remarkably, **FAI** was more effective in reducing ROS levels compared to either drug alone and comparable to their combined use. Subsequently, the ability of **FAI** to mitigate mitochondrial oxidative damage was assessed by monitoring changes in the mitochondrial membrane potential (MMP). Reduced membrane potentials prevent the accumulation of JC-1 dye in the mitochondrial matrix, resulting in green fluorescence, whereas higher membrane potentials are represented by the presence of red fluorescence associated with the polymer. It was found that **FAI** significantly reduced the levels of mitochondrial oxidative stress, outperforming the effects of the individual drugs and slightly surpassing their combined efficacy (Figure 4B).

### 2.6. The Protective Influence of ***FAI*** on LPS-Treated RAW264.7 Cells 

**Figure 5:**
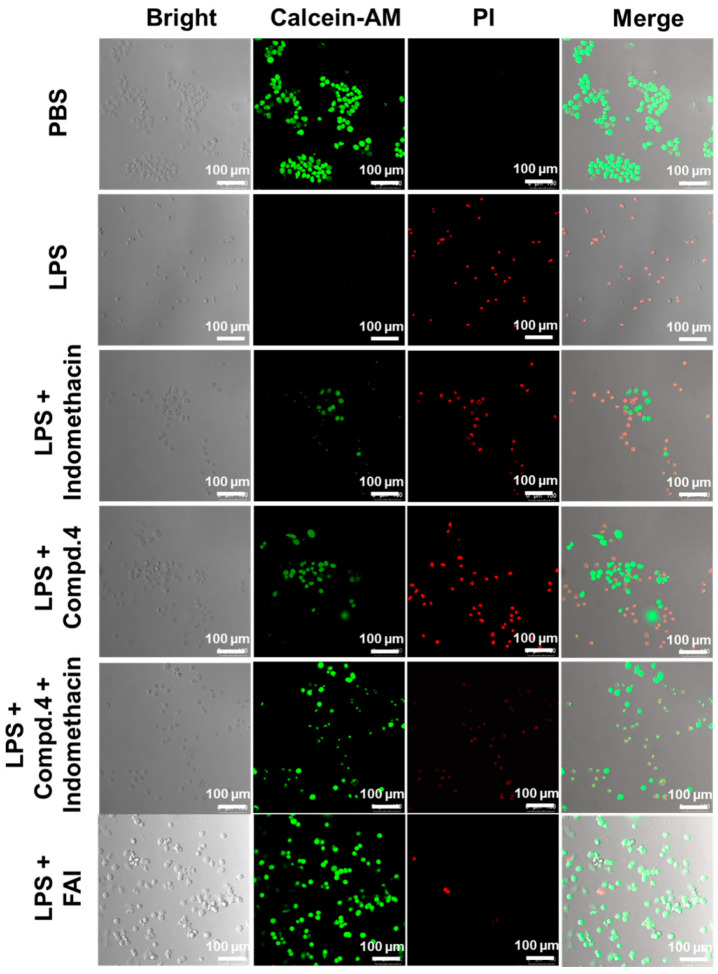
The protective effects of indomethacin, Compound **4**, a combination of indomethacin and Compound **4**, and **FAI** on LPS-treated RAW264.7 cells were evaluated by Calcein-AM/PI assay (green and red fluorescence). Green channel: λex = 490 nm, λem = 510–550 nm; red channel: λex = 535 nm, λem = 590–610 nm; scale bar = 100 μm.

As illustrated in Figure 5, the results of the live/dead cell staining of RAW264.7 cells indicate a significant reduction in the number of yellow-green Calcein-AM-labeled live cells and a significant increase in red-stained dead cells in the LPS-treated group compared to the blank group. However, the separate administration of indomethacin or Compound **4** did not result in any substantial improvement. Interestingly, the simultaneous administration of both drugs resulted in a more pronounced treatment effect. Particularly noteworthy is the marked protective effect exhibited by the prodrug **FAI** against LPS-induced RAW264.7 cells.

### 2.7. Activity and Imaging of ***FAI*** In Vivo

**Figure 6:**
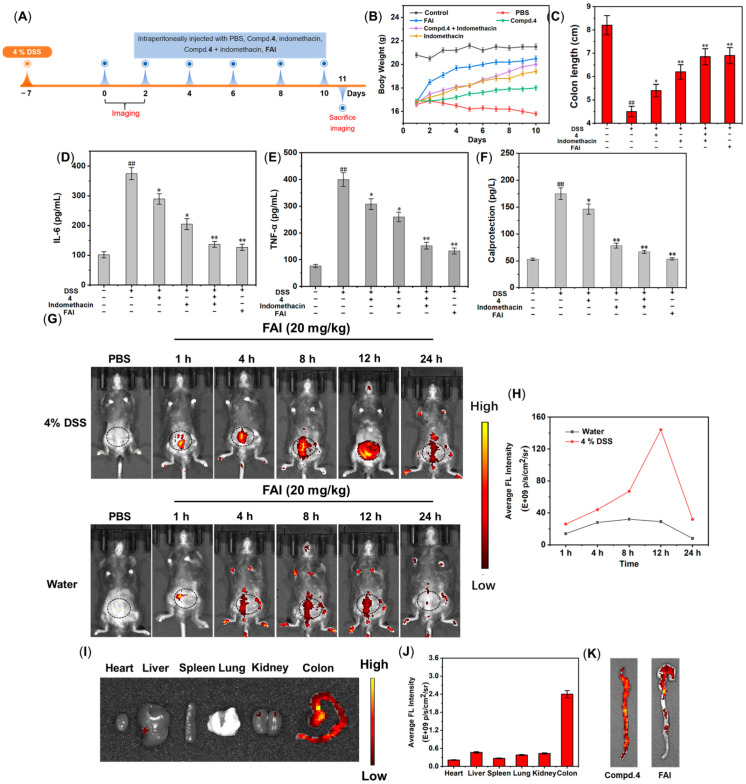
(**A**) Schematic diagram showing the timing and frequency of administration of **FAI**. (**B**) The daily weight changes in the mice in each group after drug administration. (**C**) Corresponding changes in the lengths of the colon. (**D**) The influence of each treatment on the expressions of IL-6 (**D**), TNF-α (**E**), and calprotectin (**F**) proteins in the colon tissue of UC mice. (**G**) Fluorescence imaging of mice from the normal and UC groups after intraperitoneal injection of **FAI** (20 mg/kg) in terms of time point (1, 4, 8, 12, and 24 h). (**H**) Quantification of the average fluorescence intensity of (**G**). (**I**) Fluorescence images of ex vivo organs from colitis mouse models following **FAI** administration. (**J**) Quantification of the average fluorescence intensity of major organs according to (**I**). (**K**) Ex vivo fluorescence imaging of mice treated with Compound **4** and **FAI** after intraperitoneal injection. ## *p* < 0.01 compared to the control group; * *p* < 0.05 and ** *p* < 0.01 compared to the PBS group.

The protective effects of **FAI** against DSS-induced acute colitis were also evaluated in vivo. C57BL/6 mice were randomly assigned to six groups. The normal group was treated with water, while the other groups received a 4% solution of DSS for 7 days to establish a model of acute colitis (Figure 6A). Subsequently, PBS, Compound **4**, indomethacin, Compound **4** + indomethacin, and **FAI** were injected intraperitoneally on days 0, 2, 4, 6, 8, and 10, respectively, and the mice were sacrificed and imaged on days 0–2 and 11. During the course of treatment, the daily weight fluctuations of the mice were recorded, and the fecal characteristics and presence of occult blood were observed to evaluate the severity of the colitis. As shown in Figure 6B,C, the weight of the mice in each group increased in comparison to that in the control group, with the most significant increase observed in the **FAI** group. These results indicate that the intestinal absorption in the colitis mice recovered gradually after **FAI** treatment; this was confirmed by measurements of colon length, in which the mean length of the colon in the **FAI**-treated group was 6.90 ± 0.34 cm, significantly longer than that observed in the model group (4.50 ± 0.22 cm) and closer to that of the normal group. Furthermore, relative to the control group, the model group showed a significantly increased expression of IL-6, TNF-α, and calprotectin in the colon tissues (Figure 6D–F), while the expression of IL-6, TNF-α, and calprotectin in the colon tissues of the treated mice was reduced in comparison to the model group, with the reduction being most significant in the **FAI** group and the combined treatment group, where they approached normal levels. In vivo imaging of **FAI** was undertaken by intraperitoneal injection of all mice with **FAI,** followed by fluorescence imaging at various time points. The model group showed a significant increase in fluorescence compared to the normal group, reaching a peak at 8 h with a fluorescence intensity approximately five times higher than that of the normal group, indicating the ability of the probe to visualize azo reductase upregulation in colitis (Figure 6G,H). Subsequently, the colon, heart, liver, spleen, lungs, and kidneys of the mice in the **FAI** group were harvested and imaged using fluorescence imaging on days 0–2 (Figure 6I). No obvious fluorescence signal was detected in other organs apart from the colon tissue (Figure 6J), suggesting that azo reductase was mainly distributed in the inflammatory sites of the colon, and that **FAI** could be used as an indicator for studying colitis and similar diseases. On day 11, dissection of the colon tissues from each group showed that the fluorescence in the **FAI** group was markedly lower than that in the Compound **4** group, indicating that the **FAI** treatment was more effective than that of Compound **4**, and led to decreased azo reductase levels (Figure 6K).

### 2.8. H&E and iNOS Immunofluorescence Staining

**Figure 7:**
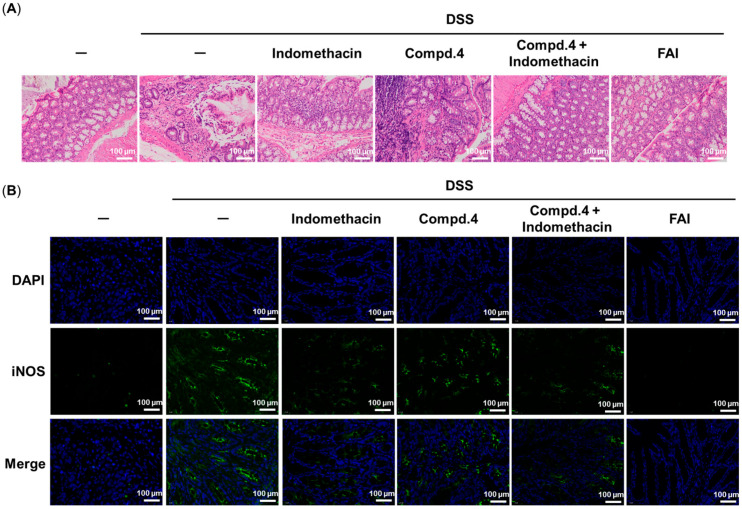
(**A**) H&E-stained sections of colon tissues from the different groups. (**B**) The expression of iNOS in colonic tissue was detected by immunofluorescence staining. Scale bar = 100 μm.

Furthermore, colonic tissues were assessed using hematoxylin and eosin (H&E) staining in each group. As depicted in Figure 7A, the mucosa of the colonic tissue in the control group appeared intact, with cells arranged in a regular pattern. Conversely, in the acute UC group, the mucosa of the colonic tissue was damaged, with the visible presence of inflammatory cells in the subepithelial layer. In contrast, both the **FAI** group and the combined treatment group showed a significant reduction in the number of inflammatory cells, with the mucosa of the colonic tissue appearing intact. Furthermore, the therapeutic efficacy of **FAI** was further verified through iNOS immunofluorescence staining. The results revealed a significant reduction in green fluorescence, marked by iNOS, nearly returning to normal levels. In summary, **FAI** demonstrates promising potential as a potent drug for the treatment of acute UC.

### 2.9. In Vivo Biosafety Evaluation of ***FAI***

**Figure 8:**
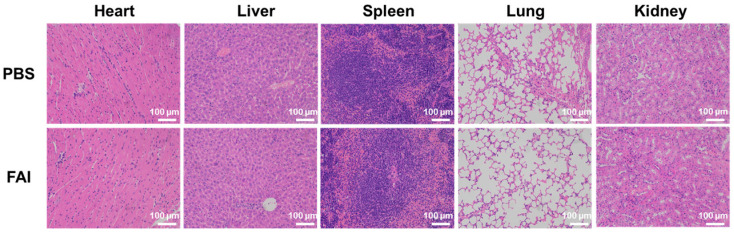
H&E staining of ex vivo tissue sections (heart, kidney, lung, liver, and spleen) from the **FAI** group.

Assessing the safety of drugs is crucial for their clinical application. To evaluate the biocompatibility of **FAI**, mice were given oral doses of high concentrations of **FAI** (500 mg/kg), and then euthanized, and their hearts, kidneys, lungs, livers, and spleens were collected for H&E staining. As illustrated in Figure 8, H&E staining revealed no histopathological or pathological damage in either the PBS group or the **FAI** group, affirming the low toxicity and superior safety profile of **FAI**.

## 3. Materials and Methods

### 3.1. Experimental Procedures

All chemical reagents utilized in this study were commercially sourced. The solvents employed were of analytical purity, with common solvents procured from Guoyu Chemical Reagents Co., Ltd. (Shanghai, China). The ^1^H NMR spectra were recorded on a 300 MHz Bruker AV spectrometer, while the ^13^C NMR spectra were recorded on a 600 MHz Bruker AV spectrometer (Bruker Co., Billerica, MA, USA), using deuterated chloroform (CDCl_3_) or deuterated dimethyl sulfoxide (DMSO) as solvents, with tetramethylsilane (TMS) serving as the internal standard. The fluorescence spectra were measured using an RF-5301PC fluorescence spectrophotometer (Shimadzu, Kyoto, Japan), while the UV-visible absorption spectra were measured using a TU-1900 UV-visible absorption spectrometer (Beijing Puxi General Instrument Co., Ltd., Beijing, China). An LSM710 confocal laser scanning microscope (Zeiss, Oberkochen, Germany) was used for fluorescence imaging. High-resolution electrospray ionization mass spectrometry (HR-ESI-MS) was performed on an Agilent 6500 Q-TOF LC/MS (Agilent Technologies, Santa Clara, CA, USA) using an ESI ion source. Column chromatography was carried out using 200–300 mesh silica gel (Qingdao Ocean Chemical Co., Ltd., Qingdao, China). The reaction was monitored using TLC glass plates (Yantai Huayang New Material Technology Co., Ltd., Yantai, China). Purification of all the tested compounds was carried out to achieve a purity of at least 95% by high-performance liquid chromatography (HPLC) using a Waters 1525 binary high-performance liquid chromatography pump equipped with a Waters 2998 photodiode array detector, and XBridge-C18 analytical column (5 μm, 4.6 mm × 150 mm).

### 3.2. Synthesis Procedure of Compound ***FAI*** 

#### 3.2.1. Synthesis of Compound **3**

1-(2-hydroxy-4-methoxyphenyl)ethan-1-one (1.66 g, 1.0 eq) and 4-(dimethylamino)-benzaldehyde (1.79 g, 1.2 eq) were dissolved in anhydrous EtOH (100 mL), followed by the addition of 20 mL of a 20% KOH aqueous solution. The mixture was stirred at room temperature for 12 h, and the reaction was monitored by TLC. After completion of the reaction, the mixture was diluted with water (100 mL), and the pH was adjusted to 7 with 6N HCl and extracted with EtOAc (3 × 100 mL), after which the organic layers were combined, dried with anhydrous Na_2_SO_4_, and concentrated under reduced pressure. The residue was then purified using column chromatography on silica gel (petroleum ether/EtOAc = 6:1), yielding chalcone **3** as a red-brown solid with a yield of 68% (2.02 g). ^1^H NMR (300 MHz, CDCl_3_) δ 13.84 (s, 1H), 7.97–7.81 (m, 2H), 7.61–7.53 (m, 2H), 7.39 (d, *J* = 15.2 Hz, 1H), 6.76–6.67 (m, 2H), 6.54–6.41 (m, 2H), 3.87 (s, 3H), 3.07 (s, 6H).

#### 3.2.2. Synthesis of Compound **4**

To a solution of Compound **3** (2.97 g, 1.0 eq) in anhydrous EtOH (100 mL), 30 mL of 5% NaOH aqueous solution was added. The mixture was stirred in an ice bath with added H_2_O_2_ (35%, 9.72 g, 10.0 eq) for 30 min. The reaction was allowed to continue at room temperature for 4 h and monitored by TLC. After completion of the reaction, the mixture was diluted with water (200 mL), and 2N HCl was added to adjust the pH to 7. The solid material was precipitated, filtered, and washed with anhydrous EtOH (3 × 50 mL). The filtrate was dried under vacuum to yield a yellow solid with a yield of 52% (1.62 g). ^1^H NMR (300 MHz, DMSO-d6) δ 9.07 (s, 1H), 8.16–8.06 (m, 2H), 7.97 (d, *J* = 8.9 Hz, 1H), 7.25 (d, *J* = 2.4 Hz, 1H), 7.02 (dd, *J* = 8.9, 2.4 Hz, 1H), 6.88–6.79 (m, 2H), 3.92 (s, 3H), 3.02 (s, 6H). ESI-HRMS (*m/z*): [M + H]^+^ calculated for C_18_H_17_NO_4_: 312.1230, found to be 312.1237.

#### 3.2.3. Synthesis of Compound **7**

To a solution of (4-aminophenyl)methanol (2.0 g, 1.1 eq) in ice water (30 mL), 3.4 mL (37%) of concentrated hydrochloric acid was added and stirred. Sodium nitrite (1.17 g, 1.05 eq) was dissolved in water (8 mL), and the mixture was cooled to 0 °C before being slowly added dropwise to the above solution, with stirring for 1 h. Phenol (1.6 g, 1 eq) and K_2_CO_3_ (3.14 g, 1.4 eq) were dissolved in water (25 mL) and slowly added dropwise to the above solution while maintaining a temperature of 0–5 °C. The mixture was stirred for 2 h, and the pH was adjusted to 4–5 using a diluted acetic acid solution until an orange-red precipitate was formed. The precipitate was filtered and washed with MeOH (10 mL), and the filter cake was dried to obtain an orange-red solid with a yield of 92% (3.4 g). ^1^H NMR (300 MHz, DMSO-d6) δ 10.29 (s, 1H), 7.87–7.71 (m, 4H), 7.59–7.45 (m, 2H), 7.01–6.89 (m, 2H), 5.35 (t, *J* = 5.7 Hz, 1H), 4.59 (d, *J* = 5.7 Hz, 2H).

#### 3.2.4. Synthesis of Compound **8**

To a solution of Compound **7** (2.28 g, 1.0 eq) in acetone (50 mL), K_2_CO_3_ (2.76 g, 2.0 eq) and 1,2-dibromoethane (2.82 g, 1.5 eq) were added and stirred for 8 h under reflux, monitored by TCL. After the reaction was complete, the mixture was poured into water (200 mL) and extracted with EtOAc (3 × 100 mL). The organic layers were combined, dried with anhydrous Na_2_SO_4_, and concentrated under reduced pressure. The residue was then purified using column chromatography on silica gel (petroleum ether/EtOAc = 2:1), yielding Compound **8** as a light-yellow solid with a yield of 82% (2.75 g). ^1^H NMR (300 MHz, DMSO-d6) δ 7.95–7.77 (m, 4H), 7.57–7.43 (m, 2H), 7.23–7.12 (m, 2H), 5.37 (s, 1H), 4.60 (s, 2H), 4.50–4.35 (m, 2H), 3.93–3.78 (m, 2H).

#### 3.2.5. Synthesis of Compound **9**

To a solution of indomethacin (3.58 g, 1.0 eq) in acetone (60 mL), K_2_CO_3_ (2.76 g, 2.0 eq) and Compound **8** (3.35 g, 1.0 eq) were added and stirred for 8 h under reflux, monitored by TCL. After the reaction was complete, the mixture was poured into water (200 mL) and extracted with EtOAc (3 × 100 mL). The organic layers were combined, dried with anhydrous Na_2_SO_4_, and concentrated under reduced pressure. The residue was then purified using column chromatography on silica gel (petroleum ether/EtOAc = 1:1), yielding Compound **9** as a light-yellow solid with a yield of 85% (5.20 g). ^1^H NMR (300 MHz, DMSO-d6) δ 7.91–7.80 (m, 4H), 7.68–7.57 (m, 4H), 7.56–7.48 (m, 2H), 7.08 (dd, *J* = 9.6, 2.4 Hz, 3H), 6.93 (d, *J* = 9.0 Hz, 1H), 6.72 (dd, *J* = 9.0, 2.6 Hz, 1H), 5.38 (t, *J* = 5.7 Hz, 1H), 4.61 (d, *J* = 5.6 Hz, 2H), 4.45 (dd, *J* = 6.1, 2.7 Hz, 2H), 4.31 (dd, *J* = 5.9, 3.0 Hz, 2H), 3.84 (s, 2H), 3.73 (s, 3H), 2.21 (s, 3H).

#### 3.2.6. Synthesis of Compound **10**

To a solution of Compound **9** (0.61 g, 1.0 eq) and CBr_4_ (0.50 g, 1.5 eq) in THF (5 mL), PPh_3_ (0.40 g, 1.5 eq) was added and stirred at room temperature for 3 h, monitored by TCL. After the reaction was complete, the mixture was concentrated under reduced pressure. The residue was then purified using column chromatography on silica gel (petroleum ether/EtOAc = 6:1), yielding Compound **10** as a light-yellow solid with a yield of 73% (0.49 g). ^1^H NMR (300 MHz, DMSO-d6) δ 8.01–7.80 (m, 4H), 7.74–7.55 (m, 6H), 7.14–7.03 (m, 3H), 6.93 (d, *J* = 8.9 Hz, 1H), 6.71 (dd, *J* = 9.0, 2.5 Hz, 1H), 4.81 (s, 2H), 4.50–4.40 (m, 2H), 4.32 (dd, *J* = 5.8, 3.0 Hz, 2H), 3.83 (s, 2H), 3.73 (s, 3H), 2.21 (s, 3H).

#### 3.2.7. Synthesis of **FAI**

To a solution of Compound **4** (0.31 g, 1.0 eq) and Compound **10** (0.74 g, 1.1 eq) in DMF (5 mL), K_2_CO_3_ (0.28 g, 2.0 eq) was added and stirred for 8 h under reflux, monitored by TCL. After the reaction was complete, the mixture was poured into water (50 mL) and extracted with EtOAc (3 × 30 mL). The organic layers were combined, dried with anhydrous Na_2_SO_4_, and concentrated under reduced pressure. The residue was then purified using column chromatography on silica gel (petroleum ether/EtOAc = 4:1), yielding **FAI** as a light-yellow solid with a yield of 79% (0.72 g). ^1^H NMR (300 MHz, CDCl_3_) δ 8.20 (d, *J* = 8.9 Hz, 1H), 8.09–8.02 (m, 2H), 7.93–7.87 (m, 2H), 7.86–7.81 (m, 2H), 7.68–7.55 (m, 4H), 7.49–7.41 (m, 2H), 7.03–6.91 (m, 5H), 6.87 (d, *J* = 9.0 Hz, 1H), 6.81–6.73 (m, 2H), 6.68 (dd, *J* = 9.0, 2.5 Hz, 1H), 5.21 (s, 2H), 4.56–4.49 (m, 2H), 4.30–4.23 (m, 2H), 3.94 (s, 3H), 3.81 (s, 3H), 3.75 (s, 2H), 3.09 (s, 6H), 2.41 (s, 3H). ^13^C NMR (151 MHz, DMSO) δ 173.2, 171.0, 168.3, 164.0, 161.4, 156.8, 156.4, 156.1, 152.1, 152.0, 146.8, 140.4, 138.1, 138.0, 135.9, 134.5, 131.6, 131.0, 130.7, 130.0, 129.5, 126.7, 125.0, 122.6, 117.8, 117.3, 115.6, 115.1, 114.8, 113.1, 111.9, 111.7, 102.1, 101.0, 72.7, 66.8, 63.3, 56.5, 55.8, 29.8, 13.6. ESI-HRMS (*m/z*): [M + H]^+^ calculated for C_52_H_45_ClN_4_O_9_: 905.2948, found to be 905.2975.

### 3.3. UV and Fluorescence Spectra

Compound **4** was dissolved separately in different solvents (DCM, DMSO, EA, and THF) to prepare a 1 mmol/L compound stock solution. Compound **4** was then diluted to 20 μM, and its UV absorption spectrum was measured in each solvent. The fluorescence emission spectrum was then recorded under the excitation wavelength equivalent to the maximum absorption wavelength of different solvents. The emission wavelength was recorded between 500 and 600 nm. Different volumes of DMSO were diluted with water to prepare DMSO/water (DMSO/H_2_O) solutions of varying concentrations, and the AIE effect of the probe was determined. The excitation slit and emission slit were both adjusted to 5 nm.

### 3.4. Characteristics of the Spectral Response of ***FAI*** to Azo Reductase

**FAI** was dissolved in DMSO to prepare a 1 mmol/L compound stock solution. The **FAI** stock solution (40 μL) was then added to PBS (0.1 mol/L, pH = 7.4), followed by the addition of a certain volume of azo reductase. The mixture was diluted using PBS to a final volume of 2 mL to obtain a mixed test solution containing 20 μmol/L **FAI** and a concentration of 0–0.45 μg/mL azo reductase. All spectral tests were performed in a PBS, 0.1 mol/L, pH = 7.4. Stock solutions for different analytes were prepared using ultrapure water. Analyte samples (1, blank; 2, K^+^; 3, Na^+^; 4, Mg^2+^; 5, ClO^−^; 6, Ca^2+^; 7, GSH; 8, Zn^2+^; 9, Fe^2+^; 10, alkaline phosphatase (ALP); 11, Cys; 12, OH^−^; 13, VcNa; 14, Cu^2+^; 15, NADPH; 16, glucose; 17, H_2_O_2_; 18, azo reductase) were added to a PBS solution containing 20 μmol/L compound **FAI** for 24 h and detected using fluorescence spectroscopy.

### 3.5. High-Performance Liquid Chromatography

An **FAI** solution (100 μM) was prepared in deionized water (5% *v*/*v* DMSO) and incubated with azo reductase (0.45 μg/mL) at 37 °C. The drug release process was monitored at different times (0, 0.5, 1, 2, 3, 6, 12, and 24 h) using an elution liquid of methanol/water (85:15, *v*/*v*). The flow rate was 1 mL/min, the column box temperature was 25 °C, and the detection wavelength was 254 nm.

### 3.6. Computational Methods of HOMO and LUMO 

ChemDraw was employed for modeling all molecular conformations, which were initially optimized using Chem3D with the MM2 force field, and then saved in the mol2 format. Further structure optimization and frequency calculations were performed using Gaussian 09 software at the B3LYP/6-311G(d,p) level with GD3BJ dispersion correction. Excited state structures and fluorescence spectra were calculated using time-dependent density functional theory (TDDFT), setting the state to 20 and using the B3LYP/def2-TZVP level with GD3BJ dispersion correction.

### 3.7. Molecular Docking

ChemDraw was used to draw the molecular structures of the selected compounds, which were then saved in the .mol format. Using Schrödinger, the azo reductase and COX 2 protein receptor files were prepared by extracting the ligands, removing the surface-crystallization water, hydrogenating the protein, and repairing the side chains. Compound 4 and **FAI** were then docked with the prepared azo reductase (PDB code: 2Z9C) and COX-2 protein (PDB code: 4OTJ) structures. The conformation with the lowest binding energy was selected as optimal. PyMOL was then used to determine the interaction mode between the target compounds and the proteins.

### 3.8. Cell Culture

RAW 264.7 cells were cultured in DMEM supplemented with 10% fetal bovine serum (FBS) and 1% antibiotics (100 U/mL each of penicillin and streptomycin, Solarbio) at 37 °C and 5% CO_2_. The cells were subsequently transferred to 35 mm cell culture dishes and incubated for 24 h to allow them to adhere to the surface.

### 3.9. Cell Cytotoxicity

Cell toxicity was assessed using MTT assays. RAW 264.7 cells were seeded in 96-well plates at a density of 10^5^ cells/mL and incubated at 37 °C with 5% CO_2_ for 12 h. Subsequently, 10 μL of various concentrations (20, 40, 80, 160, 320, and 640 μM) of Compound **4**, indomethacin, a combination of 4 + indomethacin, and **FAI** solutions were added to the respective wells, and incubation was continued for an additional 24 h. Following this, 20 μL of MTT solution (at a final concentration of 0.5 mg/mL) was added to the individual wells. The MTT solution was removed after a 4 h incubation period, and 150 μL of DMSO was added to dissolve the formazan crystals. The absorbance of individual wells was then measured at 490 nm using a microplate reader.

### 3.10. NO Assay

The culture supernatants were collected, and the NO content was measured using a NO assay kit (Beijing Solarbio Science & Technology Co., Ltd., Beijing, China), according to the manufacturer’s instructions. Absorbances were read at 540 nm in a microplate reader. The NO concentrations were calculated based on the standard curve of sodium nitrite.

### 3.11. In Vitro ROS-Scavenging Capacity of ***FAI*** 

The ability of **FAI** to clear ROS in RAW 264.7 cells was evaluated. The cells (2 × 10^5^ cells/well) were seeded into glass culture dishes and then incubated with LPS (10 μg/mL) at 37 °C for 12 h. All cells were then washed twice with PBS and incubated with DMEM containing 5 μM indomethacin, Compound **4**, a combination of indomethacin and Compound **4**, and **FAI** for 4 h. Following the removal of the media, the cells were washed once again with a fresh medium. Then, 1 mL of fresh medium with DCFH-DA (50 μM) was added to the cells. The cells were subsequently incubated at 37 °C for 30 min, followed by PBS washing (thrice), and the fluorescence images from DCF were observed under a laser confocal microscope.

### 3.12. Evaluation of Mitochondrial Membrane Potential

In brief, RAW 264.7 cells (2 × 10^5^/mL) were seeded into cell culture dishes and incubated with LPS (10 μg/mL) overnight. Then, the cells were incubated with 5 μM indomethacin, Compound **4**, a combination of indomethacin and Compound **4**, or **FAI** at 37 °C for another 24 h. JC-1 solution (10 μg/mL in DMEM) was used to replace the old medium, and the cells were incubated at 37 °C for 20 min. After incubation, the cells were separated and washed with PBS three times. A laser confocal microscope was used for evaluation and imaging.

### 3.13. Live/Dead Cell Staining

RAW 264.7 cells (2 × 10^5^/mL) were seeded into cell culture dishes and incubated with LPS (10 μg/mL) overnight. Then, the cells were incubated with 5 μM indomethacin, Compound **4**, a combination of indomethacin and Compound **4**, or **FAI** at 37 °C for another 24 h. After the treatment, 250 μL of AM-PI staining solution was added to each group and incubated for 20 min. The specimens were washed three times with PBS, and images were captured using a laser confocal microscope.

### 3.14. In Vivo Fluorescence Imaging

BALB/c mice (18–20 g, 6–8 weeks old) were purchased from the Experimental Animal Center of Nantong University. The mice were divided into 6 groups (*n* = 3). The experimental group mice received 4% DSS solution for 7 days, while the control group received distilled water. On day 7, all mice received intraperitoneal injections of indomethacin, Compound **4**, a combination of indomethacin and Compound **4**, or **FAI** (20 mg/kg). At specified time intervals, the mice were euthanized, and their hearts, livers, spleens, lungs, kidneys, and intestines were promptly collected and imaged using the IVIS Lumina system in vivo.

### 3.15. Levels of IL-6, TNF-α, and Calprotectin

Colonic tissues from all groups underwent enzyme-linked immunosorbent assay (ELISA) to determine the levels of TNF-α, IL-6, and calprotectin. Briefly, frozen tissue samples from the distal colon were homogenized in a phosphate potassium buffer (pH 6.0) containing a cocktail of protease inhibitors, followed by centrifugation at 2500 rpm and 4 °C for 5 min. The supernatants were subsequently centrifuged at 10,000 rpm and 4 °C for 10 min and the concentrations of TNF-α, IL-6, and calprotectin were measured by ELISA according to manufacturer’s guidelines. The total protein concentrations were measured using a BCA kit (Thermo Fisher, Waltham, MA, USA).

### 3.16. H&E Staining

For histological analysis of the colonic tissue, small segments (1 cm) of the distal colon were collected from the mice in the different groups and fixed in 10% formaldehyde at an ambient temperature for 24 h. The fixed tissues were embedded in paraffin blocks, cut into 5 μm-thick sections using a microtome (Reichert, Munich, Germany), and stained with hematoxylin and eosin (H&E). Images were obtained using an optical microscope (Zeiss, Axioskop, Oberkochen, Germany) for histological examination.

### 3.17. Immunofluorescence Staining

A standard protocol for immunofluorescence staining was followed. A small sample (1 cm) was collected from the distal colon of each mouse and fixed in 10% formaldehyde at room temperature for 24 h before embedding in paraffin. The embedded tissue was then cut into 5 μm-thick sections using a microtome (Reichert). Non-specific antigen-binding sites were blocked with 5% bovine serum albumin (BSA), and the sections were incubated with an anti-iNOS antibody overnight at 4 °C. The following day, the sections were washed three times with PBS and incubated with an anti-rabbit antibody for 30 min. Finally, the cell nuclei were stained with 2-(4-amino-phenyl)-6-indolocarbamidine disodium salt (DAPI) at room temperature. All images were acquired using ZEN software (Zeiss Zen 3.5) (Carl Zeiss AG, Jena, Germany).

### 3.18. Data Analysis

The data are expressed as the means ± standard deviations (SD, *n* = 3). GraphPad Prism 8 was employed for statistical analysis using one-way ANOVA and Dunnett’s multiple comparison test. A *p*-value of less than 0.05 was considered statistically significant.

## 4. Conclusions

In this study, a novel combined prodrug, **FAI**, was designed and synthesized for the diagnosis and targeted treatment of UC. Under normal conditions, **FAI** remains in an “off” fluorescence state; however, in the presence of high levels of azo reductase, it emits fluorescence, enabling the diagnosis of colitis. Moreover, **FAI** selectively released Compound **4** and indomethacin in the colon, which synergistically exert antioxidant, anti-inflammatory, and mitochondrial protective effects, with minimal toxicity to normal cells. Further in vivo assessments demonstrated that **FAI** not only possessed potent therapeutic effects against acute UC but also enabled the diagnosis of colitis within 24 h, underscoring its potential for future clinical applications.

## Figures and Tables

**Table 1:** Cytotoxic effects in RAW 264.7 cells and IC_50_ values of **FAI** against NO production in LPS-stimulated RAW264.7 cells.

Compd.	IC _50_ ± SD (μM)	
	Cytotoxicity ^a^	NO Production ^b^
**4**	897 ± 25.8	12.8 ± 1.08
**Indomethacin**	369 ± 20.3	10.3 ± 0.82
**4 + Indomethacin**	404 ± 19.8	5.9 ± 0.56
**FAI**	887 ± 29.7	5.1 ± 0.48

^a^ Concentration of compound that can inhibit 50% of cell viability. ^b^ The half-maximal inhibitory concentration (IC_50_) indicates the concentration of the compound required to inhibit NO production by 50% relative to the LPS group, with the NO release in the LPS group being 60.01 ± 0.24 μM.

## Data Availability

The data presented in this study are available in the Supplementary Materials.

## Supplementary Materials

The following supporting information can be downloaded at: https://www.mdpi.com/article/10.3390/molecules29174244/s1, Figures S1–S10: ^1^H NMRS, ^13^C NMRS and HRMS of **FAI**, Figure S12: Fluorescence emission spectra of **FAI** and Compound **4** in the absence of azo reductase, Figure S13: Fluorescence imaging in RAW264.7, Table S1: Photophysical properties of Compound **4** and **FAI** in different solvents.
