# Supplementary material for: Design and Synthesis of an Azo Reductase Responsive Flavonol–Indomethacin Hybrid Used for the Diagnosis and Treatment of Colitis
Source: Molecules. 2024 Sep 6;29(17):4244. doi: 10.3390/molecules29174244 (PMC11397019; doi:10.3390/molecules29174244)
Supplement: Supplementary file 1 [file molecules-29-04244-s001.zip › molecules-3074483-supplementary.pdf]

Supplementary Materials:

^{1}H NMRS, ^{13}C NMRS and HRMS of **FAI**.

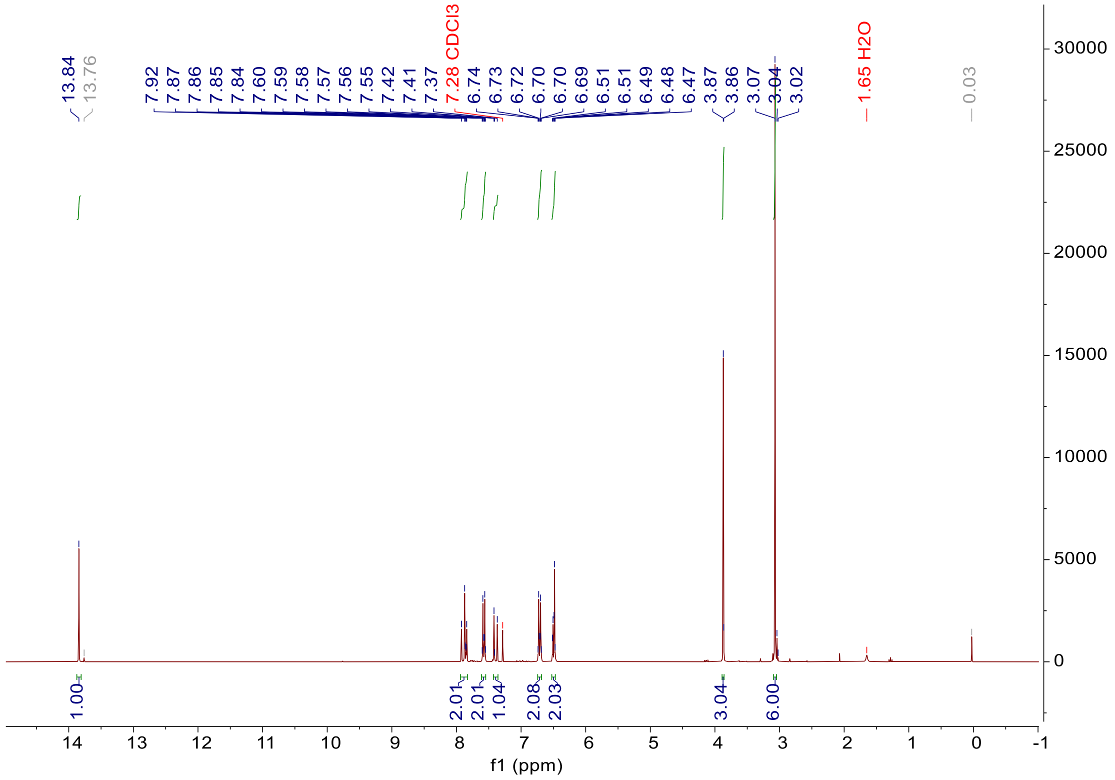

Figure S1. ^{1}H NMR spectrum (300 MHz, $CDCl_3$, 298 K) of compound **3**.

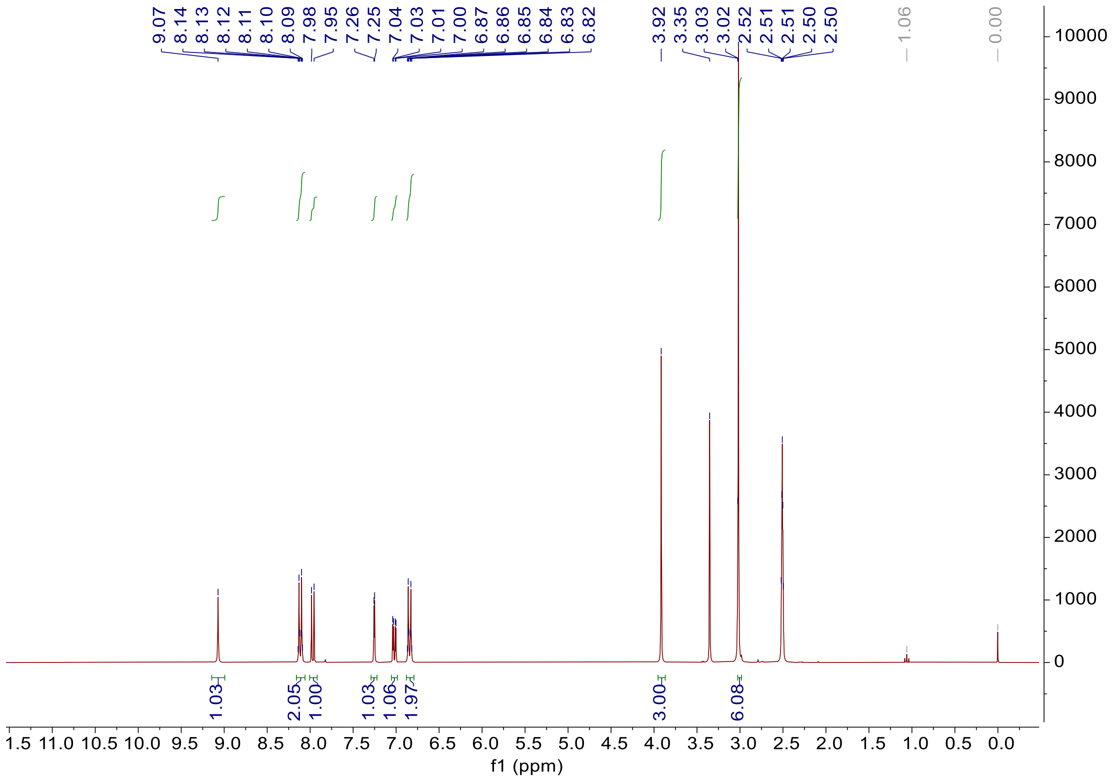

Figure S2. ¹H NMR spectrum (300 MHz, DMSO-*d6*, 298 K) of compound **4**.

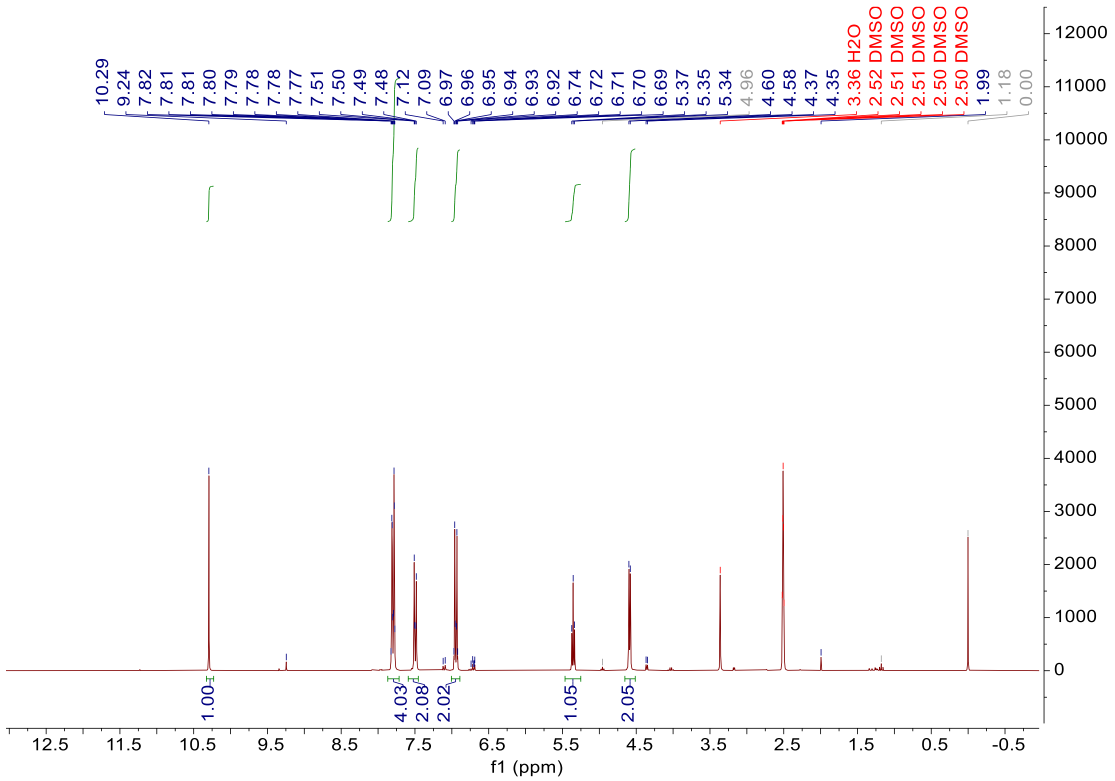

Figure S3. ¹H NMR spectrum (300 MHz, DMSO-*d6*, 298 K) of compound **7**.

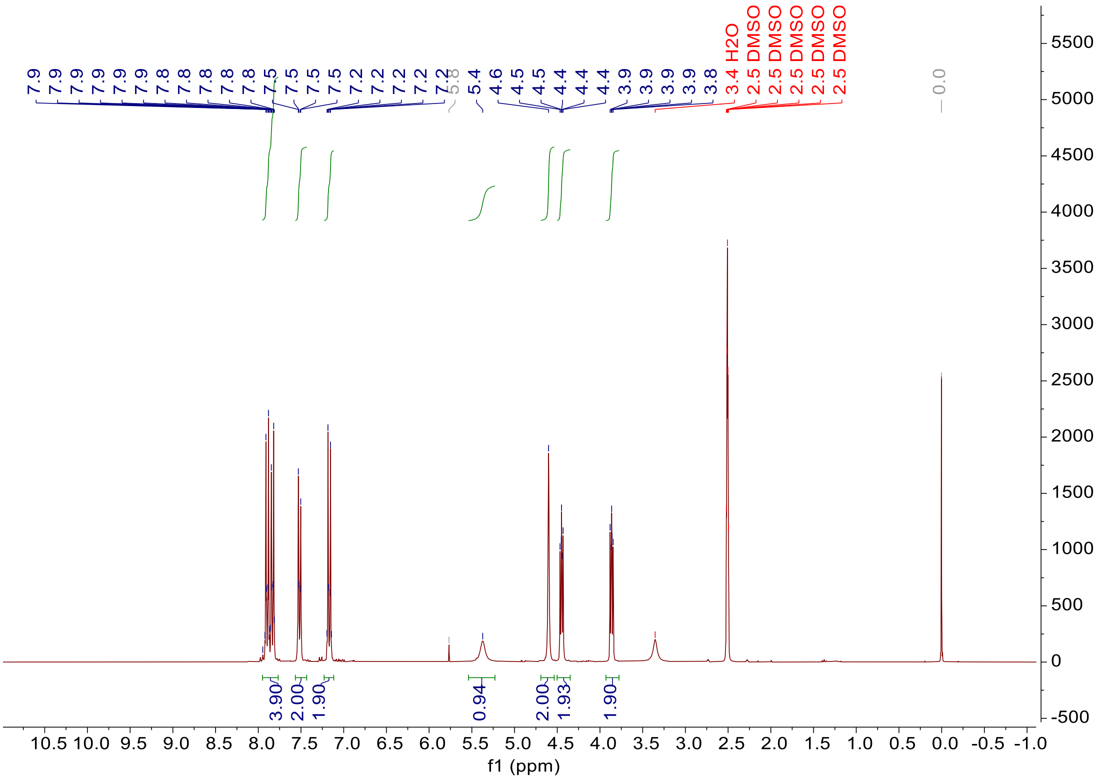

Figure S4. ^{1}H NMR spectrum (300 MHz, DMSO-*d6*, 298 K) of compound **8**.

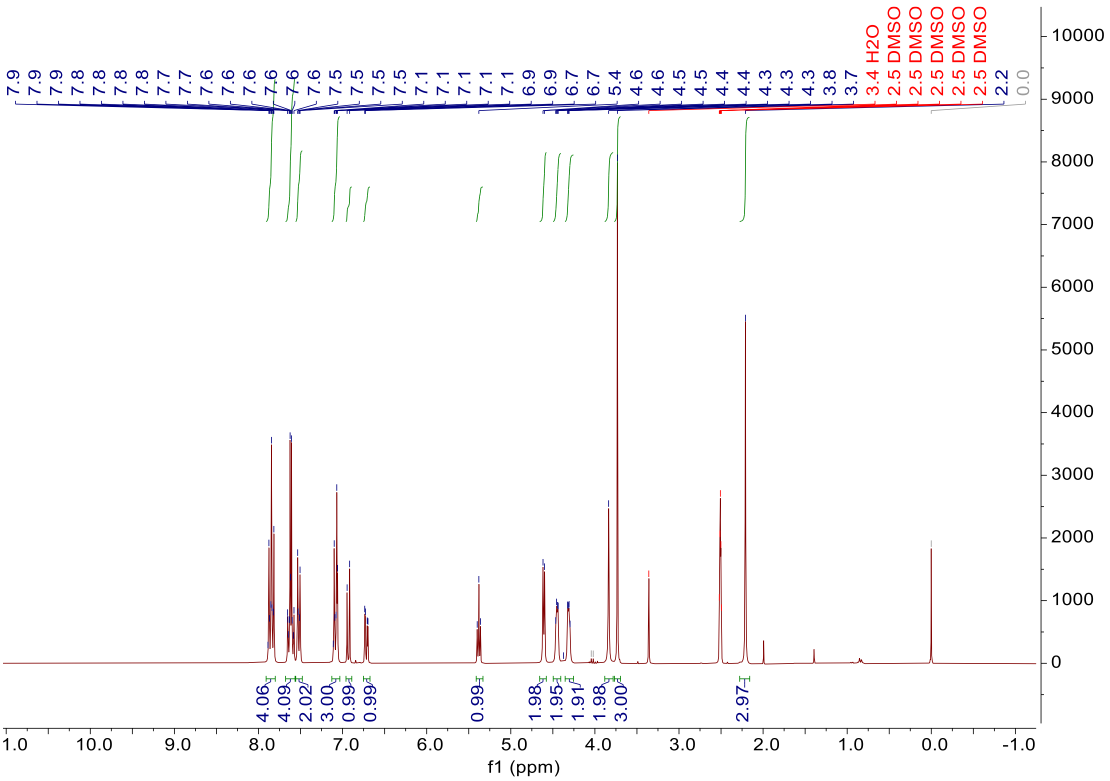

Figure S5. ^{1}H NMR spectrum (300 MHz, DMSO-*d6*, 298 K) of compound **9**.

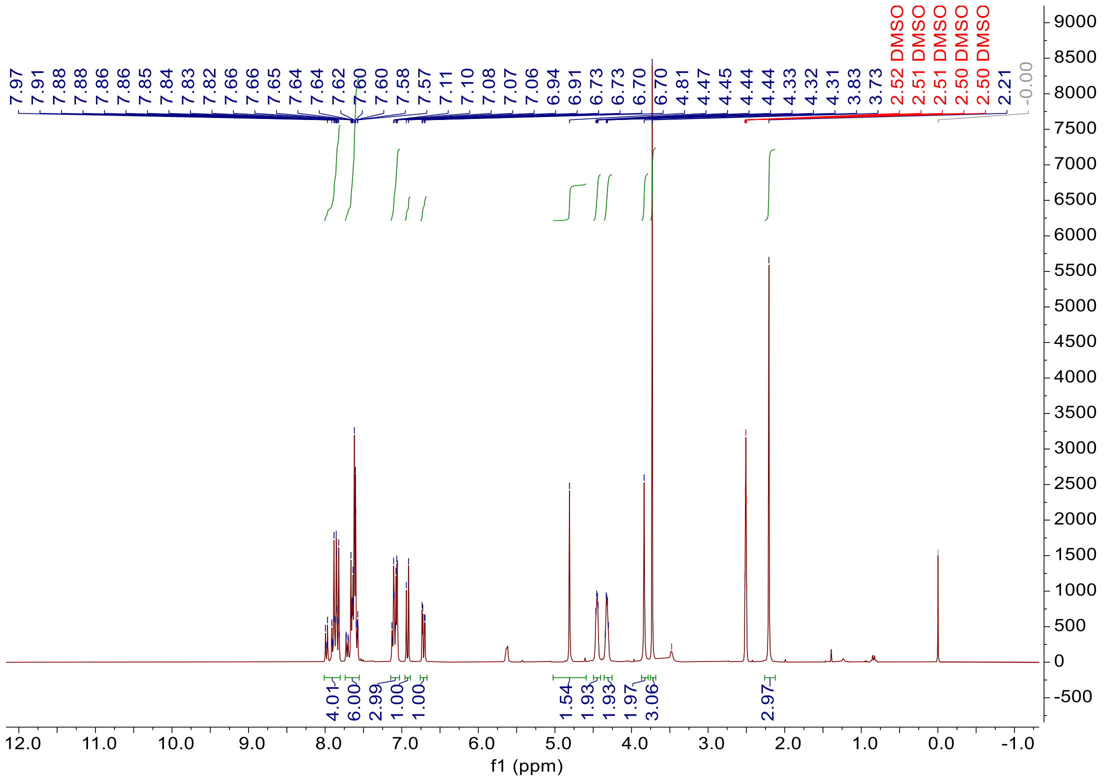

Figure S6. ^{1}H NMR spectrum (300 MHz, DMSO-*d6*, 298 K) of compound **10**.

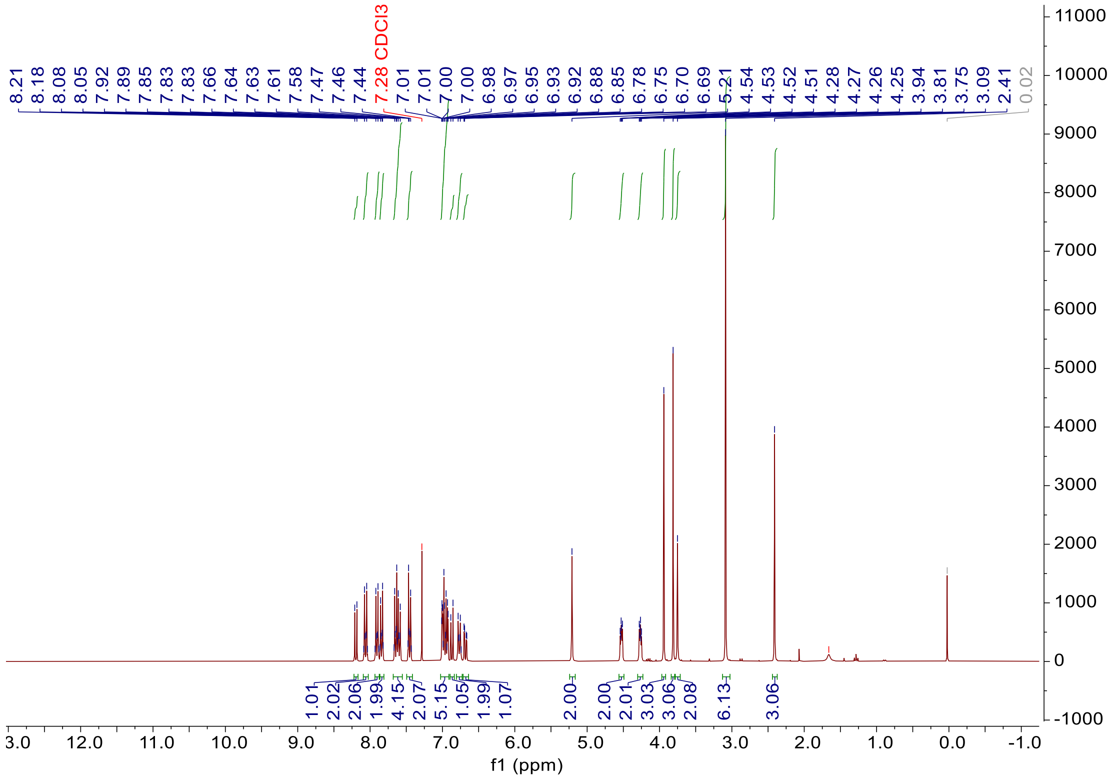

Figure S7. ^{1}H NMR spectrum (300 MHz, $CDCl_3$, 298 K) of compound **FAI**.

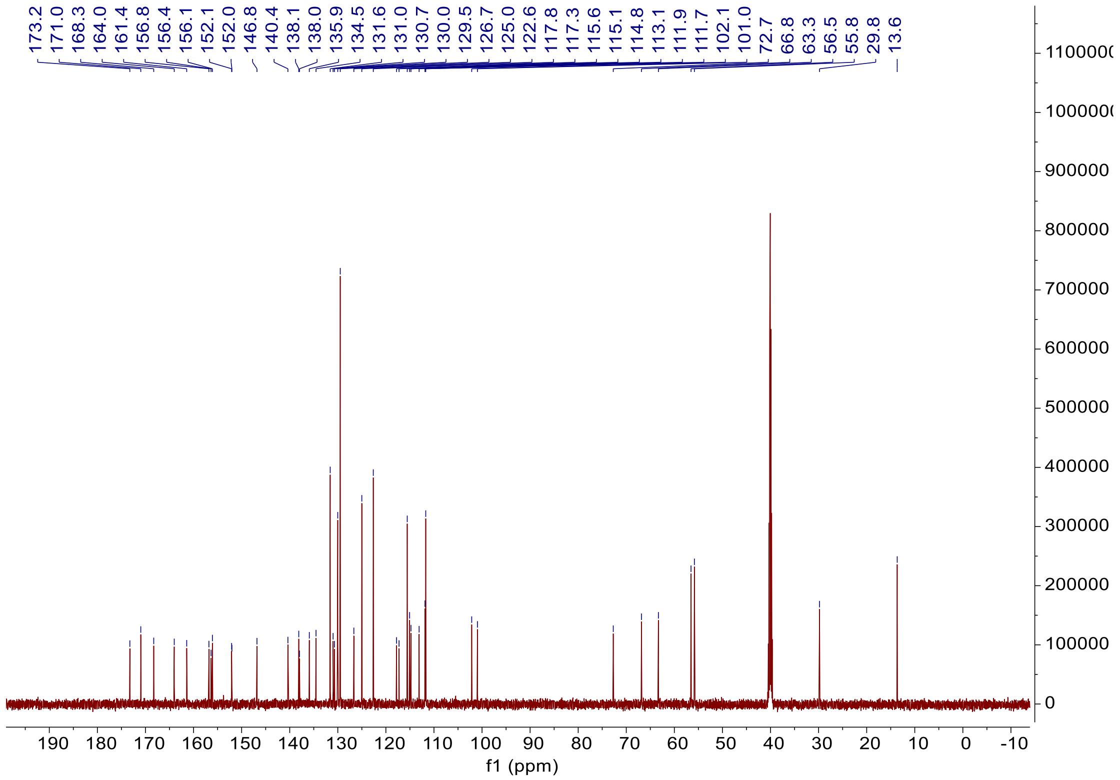

Figure S8. ^{13}C NMR spectrum (101 MHz, DMSO-*d6*, 298 K) of compound **FAI**.

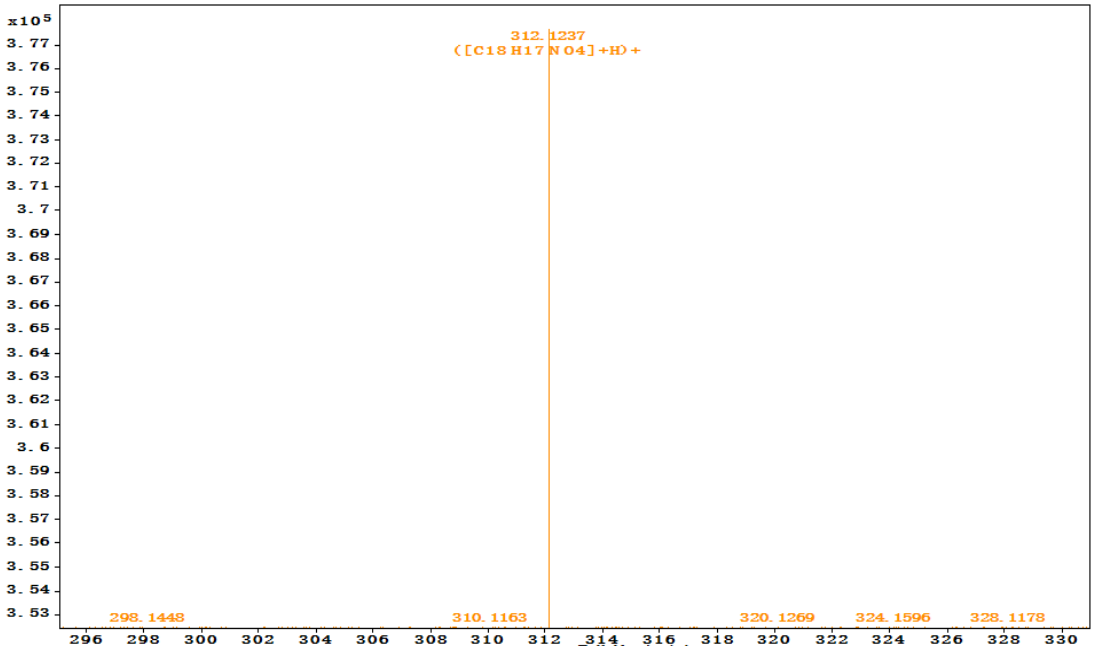

Figure S9. HRMS of compound **4**.

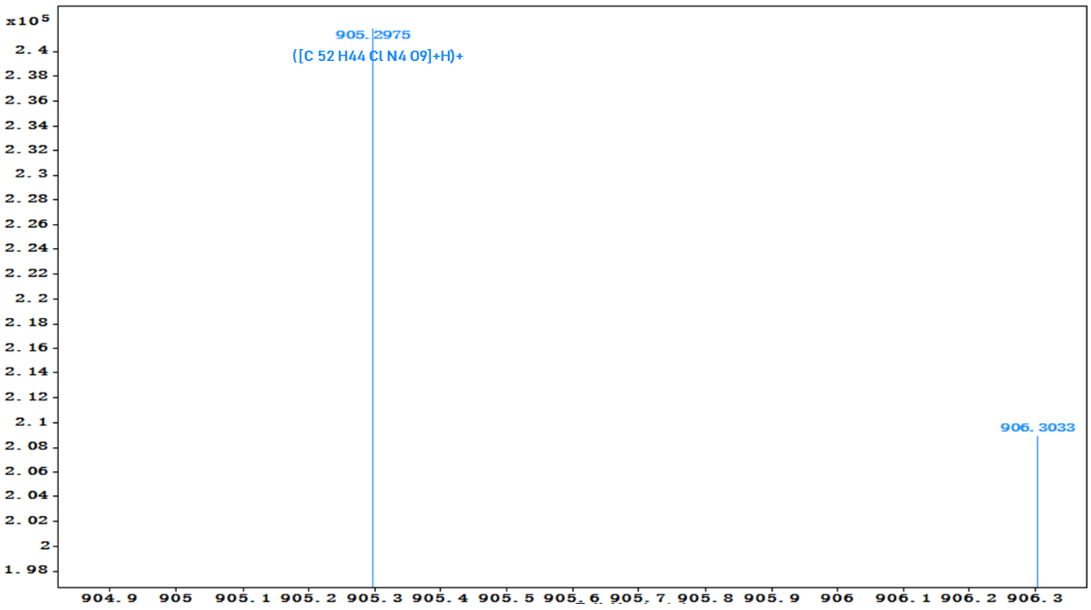

Figure S10. HRMS of compound **FAI**.

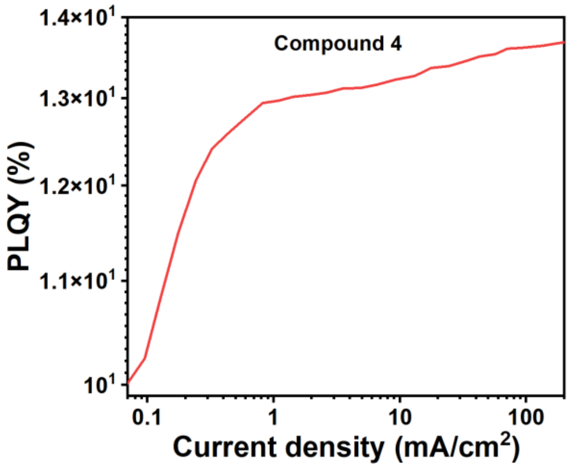

Figure S11. The absolute fluorescence quantum yield of compound 4 was measured by integrating hemispheres.

Table S1. Photophysical properties of compound 4 and FAI in different solvents.

Compd.	Solvent	λex (nm)	λem (nm)	ε [M^{-1} cm^{-1}]	Φ_{FL} (%)
**4**	DMSO	450	570	27400	13.6
	DCM	404	570	31000	N.D
	EA	390	581	30150	N.D
	THF	390	586	40500	N.D
**FAI**	DMSO	360	588	42450	< 0.1
	DCM	365	573	23500	N.D
	EA	368	580	47250	N.D
	THF	366	578	35000	N.D

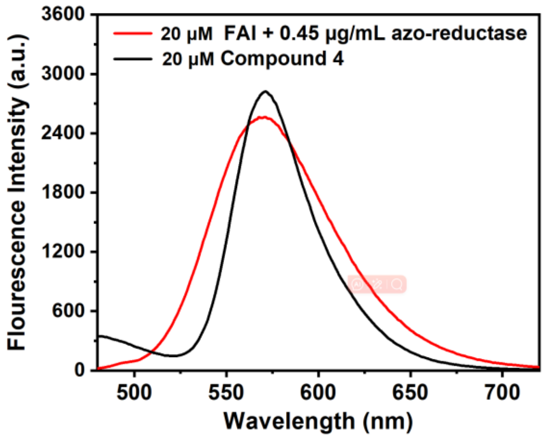

Figure S12. Fluorescence emission spectra of **FAI** and compound **4** (20 µM) in the absence of azo reductase (0.45 µg/mL).

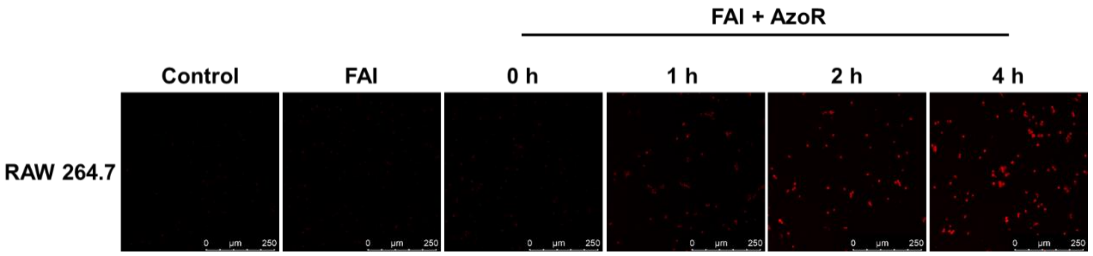

Figure S13. Fluorescence imaging in RAW264.7. First column, control; second column, images of RAW264.7 treated with **FAI** (20 µM) for 4 h; Images of RAW264.7 incubated with AzoR (0.4 µg/mL) and NADH (100 µM) for 24 h, and finally incubated with **FAI** (20 µM) for 0 h (third column), 1 h (fourth column), 2 h (fifth column) and 4 h (sixth column).
